# New spirometry recommendations from the Brazilian Thoracic Association - 2024 update

**DOI:** 10.36416/1806-3756/e20240169

**Published:** 2024-12-30

**Authors:** André Luís Pereira de Albuquerque, Danilo C Berton, Eloara Vieira Machado Ferreira Álvares S Campos, Fernando José Pinho Queiroga-Júnior, Alfredo Nicodemos Cruz Santana, Bruno de Moraes Santos Wong, Diane Rezende Batista, Felipe Xavier de Melo, Fernando Moacyr Fragoso Didier-Neto, João Adriano de Barros, João Marcos Salge, José Alberto Neder, Juliane Penalva Costa Serra, Larissa Rego Voss, Marcelo Bicalho de Fuccio, Maria Raquel Soares, Mariana Lafeta Lima, Paulo Roberto Araújo Mendes, Roberto Rodrigues-Junior, Saulo Maia D’Avila Melo, Sílvia Carla Sousa Rodrigues, Thamine Lessa, Carlos Alberto de Castro Pereira, Helen Moreira Coutinho

**Affiliations:** 1. Divisão de Pneumologia, Instituto do Coração - InCor - Hospital das Clínicas, Faculdade de Medicina, Universidade de São Paulo, São Paulo (SP) Brasil.; 2. Coordenação de Pneumologia, Rede D’Or, São Paulo (SP) Brasil.; 3. Unidade de Fisiologia Pulmonar, Hospital de Clínicas de Porto Alegre, Universidade Federal do Rio Grande do Sul, Porto Alegre (RS) Brasil.; 4. Disciplina de Pneumologia, Departamento de Medicina - EPM-Unifesp - Escola Paulista de Medicina, Universidade Federal de São Paulo, São Paulo (SP) Brasil.; 5. Setor de Função Pulmonar e Fisiologia Clínica do Exercício - SEFICE - e Doenças da Circulação Pulmonar, Escola Paulista de Medicina, Universidade Federal de São Paulo, São Paulo (SP) Brasil.; 6. Disciplina de Pneumologia, Faculdade de Ciências Médicas, Universidade de Pernambuco - UPE - Recife (PE) Brasil.; 7. Programa de Pós-Graduação Strictu Sensu, Fundação De Ensino E Pesquisa Em Ciências Da Saúde - ESCS/FEPECS - Brasília (DF) Brasil.; 8. Laboratório de Função Pulmonar Avançada, Hospital Sírio-Libanês, Brasília (DF) Brasil.; 9. Disciplina de Pneumologia, Curso de Medicina, Universidade do Sul de Santa Catarina - UNISUL - Palhoça (SC) Brasil.; 10. Laboratório de Função Pulmonar, Prefeitura de São José, São José (SC) Brasil.; 11. Divisão de Pneumologia, Faculdade de Medicina de Botucatu, Universidade Estadual Paulista - UNESP - Botucatu (SP) Brasil.; 12. Serviço de Pneumologia, Hospital Sírio-Libanês, Brasília (DF) Brasil.; 13. Serviço de Pneumologia, Hospital Universitário de Brasília, Universidade de Brasília - HUB-UnB - Brasília (DF) Brasil.; 14. Hospital Sírio-Libanês, São Paulo (SP) Brasil.; 15. Hospital Israelita Albert Einstein, São Paulo (SP) Brasil.; 16. Hospital do Coração - HCor - São Paulo (SP) Brasil.; 17. Faculdade de Medicina, Universidade Federal do Paraná, Curitiba (PR) Brasil.; 18. Laboratório de Função Pulmonar, Complexo Hospital de Clínicas, Universidade Federal do Paraná, Curitiba (PR) Brasil.; 19. Laboratório de Função Pulmonar, Hospital Nossa Senhora das Graças, Curitiba (PR) Brasil.; 20. Pulmonary Function Laboratory and Respiratory Investigation Unit, Division of Respirology, Kingston Health Science Center & Queen’s University, Kingston (ON) Canada.; 21. Serviço de Pneumologia, Hospital da Bahia, Salvador (BA) Brasil.; 22. Ambulatório de Doenças Pulmonares Intersticiais, Hospital Santa Izabel, Santa Casa da Bahia, Salvador (BA) Brasil.; 23. Serviço de Pneumologia, Hospital Santa Izabel, Santa Casa da Bahia, Salvador (BA) Brasil.; 24. Serviço de Pneumologia, Hospital Cárdio Pulmonar, Salvador (BA) Brasil.; 25. Centro de Fibrose Cística de Adultos, Hospital Júlia Kubitschek, Fundação Hospitalar do Estado de Minas Gerais - FHEMIG - Belo Horizonte (MG) Brasil.; 26. Faculdade de Ciências Médicas. Universidade de Alfenas - UNIFENAS - Belo Horizonte (MG) Brasil.; 27. Setor de Função Pulmonar, Rede D’Or, São Paulo (SP) Brasil.; 28. Serviço de Pneumologia, Hospital de Clínicas, Universidade Estadual de Campinas - UNICAMP - Campinas (SP) Brasil.; 29. Disciplina de Pneumologia, Faculdade de Medicina do ABC, Santo André (SP) Brasil.; 30. Laboratório de Função Pulmonar, Faculdade de Medicina do ABC, Santo André (SP) Brasil.; 31. Departamento de Medicina, Universidade Tiradentes, Aracaju (SE) Brasil.; 32. Laboratório de Função Pulmonar, Instituto de Assistência ao Servidor Público Estadual de São Paulo Francisco Morato Pereira - IAMSPE-FMO - São Paulo (SP), Brasil.; 33. Serviço de Pneumologia e Laboratório de Função Pulmonar, Hospital do Servidor Público Estadual de São Paulo - HSPE-IAMSPE - São Paulo (SP) Brasil.; 34. Laboratório de Função Pulmonar, Clínica AMO - Assistência Multidisciplinar em Oncologia, Salvador (BA) Brasil.

**Keywords:** Respiratory function tests, Spirometry, Respiratory physiological phenomena, Testes de função respiratória, Espirometria, Fenômenos fisiológicos respiratórios

## Abstract

The latest pulmonary function guideline from the Brazilian Thoracic Association was published in 2002, since which there have been updates to international guidelines (mainly those from the European Respiratory Society and the American Thoracic Society), as well as new national and international publications on various aspects of the performance, interpretation, and clinical implications of spirometry. Despite those updates, a careful analysis of what applies to the reality in Brazil is essential, because there have been studies that evaluated individuals who are representative of our population and who could show responses different from those of individuals in other regions of the world. This document is the result of the work of a group of specialists in pulmonary function who evaluated relevant scientific articles that could be applicable to the population of Brazil. After the discussions, new spirometry guidelines were drawn up, covering various aspects such as its technical parameters and performance; its indications and contraindications; its interpretation; concepts of normality and their related variability; reference values; classification of functional severity; and response to an inhaled bronchodilator. Finally, the guidelines emphasize the need to always interpret spirometry results in the context of the clinical condition of the patient and of the pretest probability.

## RESUMO

A última diretriz de função pulmonar da Sociedade Brasileira de Pneumologia e Tisiologia foi publicada em 2002, sendo que nesse intervalo houve atualizações de diretrizes internacionais (principalmente da European Respiratory Society e American Thoracic Society), além de novas publicações internacionais e nacionais sobre diversos aspectos da realização, interpretação e implicações clínicas da espirometria. Apesar dessas atualizações internacionais, é fundamental uma análise criteriosa do que se aplica a nossa realidade, uma vez que há trabalhos que avaliaram justamente indivíduos representativos da nossa população e que podem ter respostas diferentes das amostras internacionais. O presente documento foi resultado do trabalho de um grupo de especialistas em função pulmonar que avaliou as publicações científicas relacionadas e sua aplicabilidade para a população brasileira. Após as discussões, foram elaboradas as novas recomendações de espirometria, abrangendo diversos aspectos como sua orientação e realização técnica, indicações e contraindicações, interpretação, conceitos de normalidade e sua variabilidade relacionada, valores de referência, classificação de gravidade funcional e, finalmente, resposta ao broncodilatador inalatório. Por fim, foi destacada a associação dos resultados com o contexto clínico do paciente e sua probabilidade pré-teste.

## INTRODUCTION

After the 2002 publication of the Pulmonary Function Testing Guidelines of the Brazilian Thoracic Association (BTA), documents related to the technical aspects and interpretation were published by international societies-the European Respiratory Society (ERS) and the American Thoracic Society (ATS)-as were several articles on the topic by authors working in Brazil. In order to update the recommendations on pulmonary function, the BTA brought together a group of pulmonologists working in the area to evaluate the changes suggested by recent additions to the literature.

In this first document, it was decided that only spirometry would be addressed, leaving lung volumes, DL_CO_, and bronchial challenge testing for subsequent documents.

In consecutive meetings, the main topics were selected and distributed among groups of participants with a coordinator to evaluate the scientific literature and propose the BTA recommendation. Subsequently, each recommendation was discussed in detail, topic by topic, by all of the coordinators until a final conclusion was drawn.

Several statements regarding lung function are not based on objective studies; hence the importance of discussing each topic among experts in the field to build a consensus on recommendations. Another highly relevant aspect is the need to always seek to associate clinical data with the results of functional tests in the context of interpretation, especially for tests with marginal results (i.e., near-normal or with minimal alterations).

The main aspects related to the performance and interpretation of spirometry are discussed below. A text with more information is available in the supplementary material.

## TECHNICAL ASPECTS OF SPIROMETRY

### Patient orientation

#### When scheduling the test

When scheduling a spirometry test, inform the patient of which activities should be avoided prior to the test, and for how long before the test inhaled medications should be suspended.^1^ ^-^ ^3^ Suspending caffeine consumption before the test is no longer considered necessary (see the supplementary material).^4^ ^-^ ^6^ Patients should also be advised that they do not need to fast before the test.

#### Upon arrival at the pulmonary function laboratory

When a patient arrives at the pulmonary function laboratory, they should be told that spirometry is a noninvasive, safe, painless test that takes 30 min on average. A sample phrase is “the test will measure the capacity of your lungs and determine whether the values are normal or abnormal”.^2^

A respiratory questionnaire about symptoms, smoking, previous illnesses, medications, and previous surgical procedures should be completed to assist the medical team in interpreting the examination. Chart 1 represents a proposed model of such a questionnaire.^2^ ^,^ ^7^ ^,^ ^8^

**Chart 1:** Respiratory questionnaire on symptoms, history and exposure.

Respiratory questionnaire		
Smoking		
-Smoker ( )	Ex-smoker ( )	Never smoker ( )
-At what age did you start smoking regularly?		
-How many cigarettes did/do you smoke per day?		
-How long ago did you stop smoking?		

Respiratory symptoms		
-Do you wheeze?	Yes ( )	No ( )
-Do you usually have a cough?	Yes ( )	No ( )
-Do you regularly cough up phlegm?	Yes ( )	No ( )
-Do you have shortness of breath during any of the following activities?		
Intense exercise	Yes ( )	No ( )
Walking uphill	Yes ( )	No ( )
Walking on level ground	Yes ( )	No ( )
Light activities, such as taking a shower	Yes ( )	No ( )

Lung diseases		
-Do you or have you ever had any lung disease?	Yes ( )	No ( )
Which?	( ) Asthma ( ) Bronchitis ( ) Emphysema ( ) Fibrosis ( ) Tuberculosis ( ) Other:	
-Do you use any medication for your lungs?	Yes ( )	No ( )
Which?		
-Have you ever had any chest or lung surgery?	Yes ( )	No ( )
Which?	How long ago?	
-Have you ever been intubated?	Yes ( )	No ( )

Other diseases		
- Anemia?	Yes ( )	No ( )
- Do you or have you ever had any other illness? Yes ( ) No ( ) If yes, which?		
( )Heart ( )“High blood pressure” ( )Rheumatologic disease ( )Cancer ( )Neurological disease		
( )Other. Specify:		

-Do you or have you ever worked in an environment with dust, smoke, or chemicals (silica, asbestos, coal dust, wood stove smoke, etc.)? Yes ( ) No ( )		
- Specify the job:		

Adapted from Mottram et al.^7^ and Pereira et al.^8^

At this stage, demographic and anthropometric data (age, sex at birth, height, weight, and race/ethnicity) are collected. The technical details are described in the supplementary material.

#### Instructions for performing the test

The patient should be instructed to wash their hands. The sitting position is recommended over the standing position to avoid the risk of falling due to a loss of balance or syncope.^9^

The first step is to explain and demonstrate how to properly place the tube on the tongue, proper lip closure, placement of the nose clip, and the neutral head position.

Dental prostheses do not need to be removed if they are firmly fitted. However, the level of patient confidence in performing the maneuvers with a dental prosthesis in place should be taken into consideration.^10^ ^,^ ^11^ A poorly affixed or loose prosthesis could impair patient performance, and such prostheses should preferably be removed prior to the spirometry.

### Indications and contraindications

Spirometry is indicated for diagnosis, monitoring, evaluating dysfunction/disability, research, and studies, among other purposes (Chart 2).^2^ ^,^ ^3^ ^,^ ^12^

**Chart 2:** Indications for spirometry.

• Diagnosis • Functional assessment of respiratory symptoms and signs • Abnormal findings on additional tests (imaging, blood gas analysis, or pulse oximetry) • Measuring the physiological impact of a disease on the respiratory system • Prognostic (severity) assessment • Screening of individuals at risk for respiratory diseases • Preoperative risk assessment • Postoperative evaluation after thoracic surgery • Diagnosis of bronchial hyperresponsiveness on bronchial challenge testing
• Monitoring • Evaluate the effectiveness of a treatment • Assess the progression and exacerbation of respiratory diseases • Measure the effects of occupational or environmental exposure • Monitor the use of drugs with potential pulmonary toxicity
• Assessment of dysfunction and disability • Evaluating patients in a rehabilitation program • Assess risks as part of an insurance assessment or for legal reasons
• Other uses of spirometry • Research, clinical trials, and epidemiological surveys • Derivation of reference equations • Assess health status before starting risky/strenuous physical activities

Adapted from Graham et al.^2^

Most contraindications for performing spirometry are relative and depend on the assessment of the risk of complications, as opposed to the need to perform the test (Chart 3). In the forced maneuver, changes in blood pressure, including the potential increase in myocardial oxygen demand, as well as the intrathoracic, intra-abdominal, intracranial, intraocular, sinus, and middle ear pressures, can have adverse effects in some patients.^2^ ^,^ ^8^ ^,^ ^13^ Data in the literature indicate that patients with thoracic or abdominal aortic aneurysms can safely undergo spirometry if the aneurysm is stable, smaller than 6 cm, and not growing over time,^14^ such an aneurysm therefore not being considered a contraindication. An acute infection and impaired cognition are conditions that may lead to unsatisfactory patient performance and consequently the recording of values that represent an underestimation.

**Chart 3:** Contraindications for spirometry.

• Increased myocardial demand or changes in blood pressure • Acute myocardial infarction within a week • Symptomatic systemic hypotension or severe hypertension • Significant atrial or ventricular arrhythmia • Decompensated heart failure • Uncontrolled pulmonary hypertension • Acute cor pulmonale/clinically unstable pulmonary embolism • History of syncope during forced expiration/coughing
• Due to increased intracranial/intraocular pressure • Brain aneurysm • Brain surgery (within the first four postoperative weeks) • Recent concussion with persistent symptoms • Eye surgery (within the last week, or up to a month, depending on the type of surgery)
• Increases in sinus and middle ear pressures • Sinus or middle ear surgery, or infection within a week
• Due to increased intrathoracic and intra-abdominal pressure • Aortic aneurysm greater than 6 cm, or with signs of progression • Presence of pneumothorax in less than two weeks • Thoracic surgery (wait four weeks) • Abdominal surgery (wait four weeks) • Third trimester pregnancy
• Risk of infection • Respiratory infection, including tuberculosis, during the communicable period • Hemoptysis, significant secretion, oral lesions, or oral bleeding
• Impaired cognition precluding the testing • Cognition disorders/impaired concentration • Dementia syndrome

Adapted from Graham et al.^2^ and Cooper et al.^14^

### Acceptability and repeatability

#### FVC maneuver

It is recommended that at least three FVC maneuvers be performed, and more than eight attempts generally do not improve the quality of the test. Forced expiration in spirometry consists of four phases: 1) rapid and complete inspiration up to TLC; 2) expiration with a rapid and “explosive” start; 3) continuous expiration until reaching the 1-s plateau or until the maximum expiration time; and 4) new inspiration with maximum flow up to TLC. The acceptability criteria are described in Chart 4.

**Chart 4:** Acceptability criteria in spirometry.

Back-extrapolated volume ≤ 100 mL or up to 5% of FVC (whichever is greater)
Time to reach a PEF ≤ 150 ms
PEF variability ≤ 10% of the highest value obtained
No evidence of zero flow error
No coughing in the first second of expiration*
No glottal closure after the first second of expiration**
At least one of three of the following criteria at the end of the maneuver: 1. Expiratory plateau ≤ 25 mL in 1 s 2. Expiratory time ≥ 10 s 3. FVC is within the repeatability criterion in cases without a 1 s plateau^†^
No evidence of obstruction of the mouthpiece or spirometer
No signs of leakage

*Required for an acceptable FEV_1_ maneuver; in these cases, some maneuvers should be used only for FVC even if there is coughing in the first second. **Required for acceptable FVC; in these cases, some maneuvers should be used only for FEV_1_ even if there is glottic closure after the first second. ^†^Occurs when the patient cannot exhale sufficiently to sustain a plateau (e.g., children or patients with interstitial lung disease with increased elastic recoil), as long as the FVC is greater than or within the repeatability tolerance range of the highest FVC observed in the other maneuvers.

#### Proper start criteria

There should be an inspiratory pause of ≤ 2 s before the expiratory maneuver.

**Figure 1:**
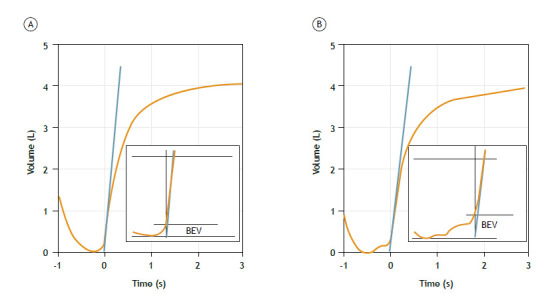
FVC and back-extrapolated volume (BEV) maneuver. The proper start of the test is with a sudden increase in flow, without hesitation. Time zero is found on the volume-time curve by a tangential line with a slope equal to the peak flow (blue line), defining the “real” time zero as the point where this line crosses the time axis. The BEV (calculated automatically in up-to-date equipment) is equal to the volume of gas expired before time zero. In this case, with an FVC of 4.0 L, the acceptable back-extrapolated limit is 200 mL (5% of FVC). The BEV was 136 mL (acceptable) for the curve A and 248 mL (unacceptable) for the curve B.

In individuals > 6 years of age, the back-extrapolated volume (BEV) should be ≤ 100 mL or up to 5% of FVC, whichever is greater; in children aged 2-6 years, the BEV should be ≤ 80 mL or 12.5% of FVC, whichever is greater (Figure 1). In 2019, the ATS recommended a BEV of ≤ 100 mL.^2^ However, a recent study demonstrated that there is little difference between a BEV of 100 mL and a BEV of 150 mL in terms of the impact on FVC and FEV_1_ repeatability.^15^ In this document, a BEV of 100 mL is considered ideal, although values up to 150 mL are considered acceptable.

**Figure 2:**
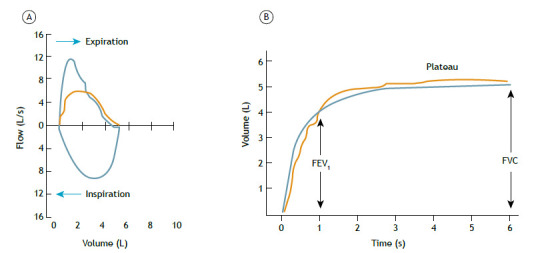
FVC maneuver: flow-volume curve (A) and volume-time curve (B). The usefulness of PEF can be seen in the flow-volume curves, with an adequate initial effort in the blue curve and a submaximal initial effort clearly demonstrated in the orange curve, which is not very evident in the volume-time curves. The detection of a constant flow close to or equal to zero at the end of the forced expiratory curve will be easily noticeable in the volume-time curve (expiratory plateau) and will be less evident in the flow-volume curve. Another desirable point is to obtain an adequate inspiratory curve with measurement of the forced inspiratory vital capacity. In this case, we observe an adequate maneuver with “meeting” of the expiratory and inspiratory curves in the blue flow-volume loop.

The time to reach a PEF, defined as the rise time between 10% and 90% of peak flow, should be ≤ 150 ms; the PEF will typically have a steep (pointed) slope but can have a flatter (rounded) slope in children, young women, and patients with neuromuscular disease. The latest ERS/ATS guidelines do not recommend repeating a PEF measure to assess the quality of the maneuver, although the study cited as a basis evaluated only elderly individuals in whom the phenomenon of effort dependence is less evident, with less influence of PEF on FEV_1_.^16^ Therefore, the recommendation of the 2002 BTA guideline to inspect and select efforts in the flow-volume curves will be maintained, discarding those with submaximal efforts, that is, PEF variability should be ≤ 10% of the highest value (Figure 2).

#### Proper end criteria

- Expiratory plateau at the end of the expiratory maneuver is defined as a volume change ≤ 25 mL in the last second of expiration, as recorded by the computer.
- For the evaluation of forced expiratory time in healthy adults, we recommend that the maneuver be stopped after a plateau has been reached, which normally occurs at around 6 s in most individuals. In children < 10 years of age, the minimum forced expiratory time is 3 s, with < 1 s being accepted in preschoolers (in this case, FEV_0.75_ or FEV_0.5_ is used in place of FEV_1_). A maximum forced expiratory time of 15 s is sufficient, because longer times generally do not alter the interpretation. We recommend accepting a forced expiratory time of 10 s as sufficient in individuals who do not achieve a plateau, given that some patients experience fatigue during longer forced maneuvers.
- The test (maneuver) should be discontinued even in the absence of a plateau if there is marked discomfort or syncope, if the subject is a child, or if there is marked restriction (for example, in some cases of muscular dystrophies), as long as the values obtained meet the repeatability criteria.
- Compare the FVC with the volume of maximum inspiration; that is, the forced inspiratory vital capacity (FIVC). It is recommended that the difference between the FIVC and FVC be < 100 mL or 5% of the FVC (whichever is greater) and that the expiratory and inspiratory curves “meet”, indicating that the FVC maneuver started from an inspiration close to TLC (Figure 2). Occasionally, in cases of obstructive disorders, the FIVC can be greater than the FVC because of the phenomenon of dynamic compression of the airways during exertion.

#### Repeatability criteria between maneuvers

Repeatability is defined as the difference between the two highest measurements obtained in different maneuvers. The grading of quality in spirometry is related to acceptability and repeatability (Chart 5).

**Chart 5:** Grading of quality in spirometry.

Grade	Maneuvers	Repeatability > 6 years	
		FVC and FEV_1_	PEF
A	≥ 3 acceptable	≤ 100 mL	≤ 10% of the highest value
B	≥ 2 acceptable	> 100 mL and ≤ 150 mL	≤ 10% of the highest value
C	≥ 2 acceptable	> 150 mL and < 200 mL	≤ 15% of the highest value
D	1 acceptable	N/A	N/A
Z	0 acceptable	N/A	N/A

N/A = not applicable.

#### *Usable parameter definition (FEV*_*1*_ and FVC)

In some cases, maneuvers that do not meet the acceptability criteria can still provide FEV_1_ and FVC values that are useful for interpretation. For example, early termination of a maneuver is not a reason to discard all data obtained, and FEV_1_ may be a valid (usable) measurement, provided that there were no artifacts during the first second of the test.

#### Slow vital capacity maneuver

In addition to the forced step, the vital capacity maneuver can be performed by obtaining the parameters vital capacity, inspiratory capacity (IC), and RV, including the possibility of calculating the FEV_1_/vital capacity (FEV_1_/VC) ratio. In this case, it is preferable that the vital capacity maneuvers be performed before the FVC maneuvers, because some patients with severe airway obstruction have a momentarily high level of functional residual capacity (FRC) after maximum inspiratory effort and a consequent drop in IC as a result of dynamic lung hyperinflation.

In spirometry, the vital capacity is typically obtained during expiration. The vital capacity maneuver should be performed in a relaxed manner (except at the end of inspiration and expiration, which should be at maximum effort); starting from TLC to RV, with the end of the test defined by a variation in volume ≤ 25 mL for at least 1 s. The IC maneuver should also be performed in a relaxed manner, with at least three stable breaths in V_T_, from FRC to TLC. Obtain at least three acceptable maneuvers (from up to eight maneuvers, if necessary) with stability of the baseline V_T_ in at least three breaths, with a difference of no more than 15% in relation to the highest value of V_T_. If stability does not occur in eight respirations, proceed to the vital capacity maneuver.

The repeatability criteria for vital capacity and CI are ≤ 150 mL or 10% of the highest value (in individuals > 6 years of age). The highest vital capacity obtained should be selected. The mean of the CI values should be obtained from curves with stability at the baseline V_T_ (otherwise, the CI should not be valued).

## CONSIDERATIONS ON THE INTERPRETATION OF SPIROMETRY

### Reference values

Reference values for lung function are those obtained in individuals who have never smoked and without current or previous cardiopulmonary or systemic diseases.^17^ They should be obtained from the same population in which the tests will be applied, as they vary widely according to the country of origin. The equations selected for the various pulmonary function tests should be included in the pulmonary function reports.

Reference values are influenced by sex, height, and age. The use of separate equations for different races or the use of a multiracial equation is of great interest at present,^18^ and more data are needed in order to make that choice for use in Brazil. Although body weight is a minor determinant of predicted values from forced spirometry, it is noteworthy that studies have excluded obese individuals. Reductions in lung volume can be found in obese individuals, the most common being reductions in RV and FRC.^19^ Despite the minimal influence that body weight has on the predicted value, it is important to identify the weight or BMI of the patient in the report, because that can facilitate the interpretation of the results.

Lung volumes and maximal expiratory flows increase progressively during childhood and typically correlate well with height. Lung function reaches peak values at 18-20 years of age in women and a bit later, at around 25 years of age, in men because of an increase in inspiratory muscle strength in the latter.^20^ Up to age 35, FVC and FEV_1_ change little, declining progressively thereafter, with survival being longer in individuals with greater lung function.^21^ Very elderly individuals who are able to perform spirometry have higher lung function values than do those who are not, and the use of derived values as a reference attenuates or abolishes the decrease in projected values; thus creating a selection bias. Therefore, it is correct to use extrapolated values to estimate predicted values in the very elderly.^22^

The expected range for lung function measurements is wide and, whenever possible, variations in lung function should be compared with values previously obtained from the same individual.

### Reference equations

In the absence of values derived from the local population, it was previously recommended that foreign equations be adopted, which resulted in large biases depending on the study chosen.^23^ A multicenter study conducted in Brazil was published in 2007.^23^ The study involved 643 White adults, 20-85 years of age, who were evaluated with computerized flow spirometers, and the results were analyzed according to rigorous criteria.

In 2012, the Global Lung Function Initiative (GLI) proposed a set of equations for universal adoption.^24^ A total of 74,187 nonsmoking individuals in 26 countries on five continents were included in equations derived by combining several studies. The quality of the curves was not assessed. In the GLI study, a new statistical model was proposed.^24^

A comparison of the GLI equation with data derived from the 2007 Brazilian sample showed that the lower limit of the predicted FEV_1_/FVC ratio is significantly lower when the GLI equation is applied.^25^ This results in it having lower sensitivity for diagnosing airflow obstruction, which can be attributed to the inclusion of several low-quality studies, widening the range of predicted values. Although the GLI committee insists on the universal adoption of these equations,^26^ this underdiagnosis of obstruction underscores the importance of using the predicted values derived from our population.

Spirometry reference values for in Black adults in Brazil were published in 2018.^27^ A comparison with predicted values derived for Whites showed lower values, especially in males, but with smaller differences than those suggested for individuals in the United States. We know that race alone might not be the only factor responsible for such a difference, because there could be socioeconomic and environmental factors that were not considered. Therefore, it is recommended that the equation specific to the Black race be reserved as an option for tests of Black individuals with borderline values, always being considered within the context of the clinical condition and the pretest probability of disease.

One large study on pediatric reference values in Brazil included children 3-12 years of age (Chart 6).^28^ As was observed in adults, the adoption of GLI equations was found to reduce the sensitivity for the diagnosis of airflow obstruction, because the lower limits for the FEV_1_/FVC ratio are also lower*.*

**Chart 6:** National reference values according to sex and age group.

Age range	Male	Female
3-12 years	Jones et al.^28^	Jones et al.^28^
13-19 years		Mallozi MC^a^
13-24 years	Mallozi MC^a^	
> 20 years		Pereira et al.^23^
≥ 25 years	Pereira et al.^23^	

a Based on Mallozi MC. Valores de Referência para espirometria em crianças e adolescentes, calculados a partir de uma amostra da Cidade de São Paulo [thesis].São Paulo: Escola Paulista de Medicina, Universidade Federal de São Paulo; 1995.

#### Lower limits

**Figure 3:**
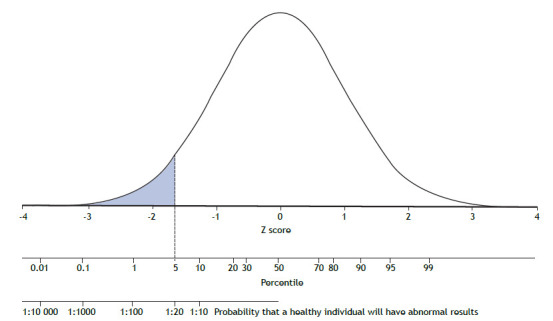
Derivation of the lower limit by the Z score and the 5th percentile of the residuals. This limit means that the result will be considered abnormal for one in every twenty healthy individuals.

Various biological variables, such as FVC, when placed in order, will follow a distribution curve known as “normal” or Gaussian. The variation of the data around the mean is assessed by measures of dispersion, the most common of which is the standard deviation (SD). The mean ± 2 SD encompasses 95.4% of the sample values (47.7% on each side of the mean). The Z score (observed value − mean ∕ SD) expresses how far, in multiples of SD, the individual is from the mean, and will be considered abnormal if less than 1.645 (Figure 3). Another way to estimate the lower limit is to determine it by the 5th percentile. When the distribution of values around the mean follows a normal curve, the 5th percentile and the Z score are very similar. In this situation, the choice of one of these two methods is irrelevant.

In statistics, if the dispersion around the regression curve is constant, the lower limit will be established by subtracting a fixed value from the predicted value. It follows that the lower limit cannot be established by a fixed percentage, such as 80% of the predicted FVC and FEV_1_ in adults, which is an outdated simplification.^29^ Ideally, the principles of clinical decision-making should be applied, requiring assessment of preclinical probability to determine the presence or absence of disease in the face of values close to the threshold of abnormality.

If the dispersion around the regression decreases proportionally as the predicted value decreases, the residuals will better fit the normal distribution by logarithmic transformation of the variables. In this situation, the lower limit is a fixed percentage and is independent of the predicted value. Spirometric values in children and flows in adults fit this model better (Chart 6).^23^ ^,^ ^27^ ^,^ ^28^

A value situated at the lower limit of normal (LLN) means that 95% of the healthy reference population has a value above this value. Therefore, this does not mean that values slightly below the LLN indicate that the individual being tested is ill. For this, the pretest probability of disease must be considered. The respiratory questionnaire is intended to assess the pretest probability. A value close to the LLN can be considered abnormal in the presence of findings indicative of the condition under investigation. Obviously, values well below the LLN have a greater positive predictive value for disease.

### Interpretation of spirometry

Appropriate interpretation of spirometry requires measurements that meet the prerequisites for technical quality. Low-quality tests should be interpreted by expressing the appropriate level of uncertainty, and the possibility that the measured values could reflect technical rather than pathophysiological deficiencies should be considered.

#### *FEV*_*1*_ /FVC ratio

Obstructive ventilatory impairment is characterized by a disproportionate reduction in maximum airflow in relation to the largest volume of air that can be expired from the lungs after a maximal inspiration. The most important parameter in identifying airflow obstruction is the reduction in the FEV_1_/FVC ratio below the 5th percentile of the predicted value (i.e., the LLN).

The definition of persistent airflow limitation as an FEV_1_/FVC ratio < 0.70 after bronchodilator use was maintained in the GOLD guideline.^30^ Although easy to remember, this criterion is controversial because it disregards the age-related physiological decline in the FEV_1_/FVC ratio, leading to underdiagnosis and overdiagnosis of obstruction in young people and the elderly, respectively. Since 2005, the ATS/ERS consensus on spirometry interpretation has defined airflow limitation as an FEV_1_/FVC ratio below the LLN.^26^ ^,^ ^31^

Several studies have compared the diagnosis of airflow limitation based on an FEV_1_/FVC ratio < 0.70 with that based on an FEV_1_/FVC ratio < LLN. In one large study, more than 11,000 patients, with a mean age of 63 years, were followed for 15 years. The definition of airflow obstruction based on an FEV_1_/FVC ratio < 0.70 was more accurate than was that based on an FEV_1_/FVC ratio < LLN in predicting respiratory-related hospitalization and death.^32^ The equation used for the LLN was that of the GLI, which explains the inferiority in comparison to the fixed limit. With the GLI equation, as previously noted, the LLN is much lower than with other equations, such as the one suggested for Brazil,^25^ which explains the lower sensitivity of the GLI equation to characterize the presence of airflow obstruction, and hence the apparent superiority of the fixed limit in the study cited above.

The GOLD guideline has also always characterized the presence of COPD by an FEV_1_/FVC ratio < 0.70 after BD use. A recent study showed that individuals with pre-BD obstruction (defined as an FEV_1_/FVC ratio < 0.70) but without post-BD obstruction had, after adjustment for other variables, a 6.2-times greater risk of developing COPD.^33^ On the basis of these data, we suggest that the diagnosis of airflow obstruction be based on a pre-BD FEV_1_/FVC ratio < LLN. It should be borne in mind that individuals with asthma can show this same type of response.

Another point of controversy concerns the interchangeable use of FVC or vital capacity as the denominator in the FEV_1_ ratio. The FVC can underestimate vital capacity because of early closure of the small airways at low lung volumes in the forced maneuver. However, there is a risk of false-positive results for airflow obstruction, because the LLN of the FEV_1_/VC ratio used in clinical practice comes from the same reference equations used in order to evaluate the FEV_1_/FVC ratio, which might not be correct. Nevertheless, there is evidence that the use of the FEV_1_/VC ratio increases the rate of individuals diagnosed with airflow obstruction presenting abnormalities consistent with airway dysfunction and a greater clinical probability of disease.^34^ ^-^ ^36^ In individuals > 70 years of age, the FEV_1_/VC ratio should be used with caution because it has been shown not to indicate a greater probability of disease or airway dysfunction.^35^ In this age group, vital capacity and FVC values differ more widely in the reference population. Studies comparing the FEV_1_/VC and FEV_1_/FVC ratios in patients, using separate predicted values but derived from the same population sample, are needed to resolve this controversy.

**Figure 4:**
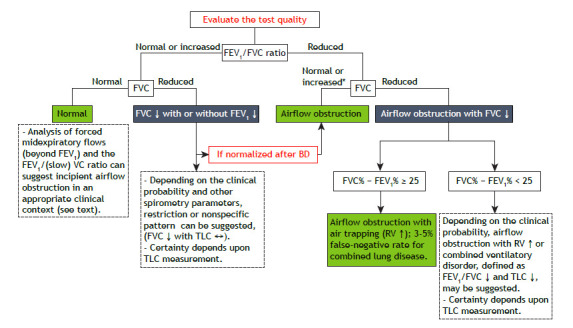
Spirometry interpretation algorithm. Increased (­), normal («) or reduced (¯) values are relative to the statistical limits of normality: ­ = above the upper limit of normal or > 95th percentile (Z score + 1.645) of the healthy population; ¯ = below the lower limit of normal or < 5th percentile (Z score − 1.645) of the healthy population. BD: bronchodilator; FVC%: FVC in percentage of the predicted value; FEV_1_%: FEV_1_ in percentage of the predicted value.

Although airflow obstruction with reduced FVC most often corresponds to increased RV (air trapping), associated restriction characteristic of combined lung disease cannot be ruled out.^37^ If it is not possible to measure TLC, a difference between FVC and FEV_1_, in % of predicted (FVC% and FEV_1_%, respectively), of ≥ 25 suggests, with a high degree of certainty,^38^ ^,^ ^39^ airflow obstruction with air trapping (Figure 4). In a test with a reduced FEV_1_/FVC ratio and reduced FVC%, with a difference between FVC% and FEV_1_% < 25, measurement of TLC is recommended to better characterize the disorder (airflow obstruction with airtrapping or combined ventilatory disorder). In 3% of cases, TLC is reduced in the presence of preserved FVC in the context of obstruction.^39^

#### Forced expiratory flows

The first change associated with airflow obstruction indicative of COPD is believed to be a slowing of the mean expiratory and end-expiratory flows on forced spirometry. Although FEF_25-75%_ is one of the most widely studied parameters, it is considered to have high intra- and inter-individual variability and a wide normal range.^40^ In many studies, that is due to inadequate derivation of predicted values, either by including tests without quality verification or by using inappropriate prediction equations, such as those derived from non-log-transformed data.^41^

Despite these limitations, a lower FEF_25-75%_ has been shown to be associated with more extensive emphysema, bronchial hyperresponsiveness, and lung hyperinflation, regardless of the FEV_1_.^42^ Other studies have shown that smokers with reduced FEF_25-75%_ and normal FEV_1_/FVC are more likely to develop COPD thereafter.^43^

Still in the search for spirometry parameters that are more representative of more distal airways, the analysis of FEF_75%_, in one study,^44^ added sensitivity to the FEV_1_/FVC ratio for the detection of airflow limitation in symptomatic patients with suspected obstructive disease and preserved FVC.

It should be noted that flows can be considered reduced in isolation only in the presence of FVC within the predicted range. A reduction in lung volume results in proportionally lower flows. In the presence of reduced FVC, a reduction in the FEF_25-75%_/FVC ratio indicates airflow limitation. This parameter is particularly important for characterizing obstruction in children with an FEV_1_/FVC ratio within the predicted range.

The ratio between FEV in 3 s and FVC^45^ and the ratio between FEV in 3 s and FEV in 6 s have been suggested as alternative measures to assess the terminal portion of the spirometric curve,^46^ but reference values are not available for the Brazilian population.

In brief, these additional parameters related to intermediate or more distal flows could be additional variables in patients with suspected obstructive disease.

#### *Reduction in FEV*_*1*_ and in FVC with no reduction in the FEV_*1*_ /FVC ratio

A reduction in FEV_1_ and in FVC with no reduction in the FEV_1_/FVC ratio is a set of findings commonly described as suggestive of restrictive lung disease (RLD), which is, however, physiologically characterized by reduced TLC. The presence of restrictive disease (e.g., fibrosing interstitial lung disease) determined from a respiratory questionnaire, an increased FEV_1_/FVC ratio (> 110% of the predicted value), with or without an FEF_25-75%_ > FVC in absolute values, and a convex expiratory flow-volume curve increase the likelihood of restriction.^47^ Because vital capacity constitutes the majority of TLC, FVC < 50% of predicted is most commonly observed when TLC is reduced.^47^

In early obstruction, however, collapse of the small airways can reduce FVC and increase RV before the FEV_1_/FVC ratio decreases, creating the possibility of a “pseudo-restrictive” pattern. The presence of significant variation in spirometric parameters after bronchodilator use confirms that. The clinical context, together with an analysis of the spirometry results, can help define which of these possibilities we are testing. If uncertainty remains, measurement of TLC is recommended (Figure 4).

A reduced FEV_1_/FVC ratio with increased FVC or FEV_1_ within the normal range can be due to dysanapsis (defined as a mismatch between the growth of the airways and that of the lung parenchyma), or more commonly, it can result from greater airway compression in younger men with expiratory muscles at a mechanical advantage due to larger lung volume (“variant of normal”). Whether this pattern represents airflow obstruction will depend on the clinical likelihood of obstructive disease and possibly on the results of additional functional testing.^48^ ^,^ ^49^

### Severity classification

In assessing the severity of functional impairment, tests should ideally be able to assess its relationship with survival, quality of life, symptom intensity, and the probability of clinical worsening, hospitalization, or both. 

However, because various diseases can manifest as the same respiratory disorder, the magnitude of functional limitation does not necessarily reflect the same prognosis among them. Factors other than lung function, such as anemia, sarcopenia, and heart disease, can influence the clinical outcomes of respiratory diseases. Traditionally, the consensuses on lung function from the main international societies have used studies that evaluated several functional parameters,^3^ ^,^ ^26^ especially FEV_1_ and FVC, to predict mortality in the most representative lung diseases, such as COPD for airflow obstruction and idiopathic pulmonary fibrosis (IPF) for RLD.

### Classification: cutoff points

#### *Obstruction: FEV*_*1*_ *and the FEV*_*1*_ */FVC ratio*

Studies conducted in the 1970s and 1980s identified post-BD FEV_1_% as the functional parameter that best correlates with survival in COPD, showing an association between progressive functional decline and mortality.^50^ ^-^ ^52^ In various studies, FEV_1_ < 50% of predicted values have been shown to correlate with worse survival. Among patients with an FEV_1_ < 30% of predicted, the mean 5-year survival rate was found to be 25%. One study evaluated different cutoff values for severity classification in 611 individuals with COPD^53^ and found that those proposed in the 1997 British Thoracic Society guidelines^54^ and adopted by the BTA in 2002^3^ had greater sensitivity and lower specificity for predicting 5-year mortality when compared with the 2023 GOLD and 1995 ATS scales (Table S1, supplementary material).^55^ More recently, a study involving 3,665 patients with COPD identified superiority of other cutoff points (≥ 70%, 56-69%, 36-55%, and ≤ 35% of predicted) to discern different levels of mortality in 5 years when compared with the GOLD cutoff points (≥ 80%, 50-79%, 30-49%, and ≤ 30% of predicted) and the proposed BODE cutoff points (≥ 65%, 50-64%, 36-49%, and ≤ 35% of predicted).^56^ Finally, a new proposal for a classification system, using the Z score, was established because it correlated with the FEV_1_% adopted by the ATS/ERS in 2005 and currently adopted in the 2022 international update.^26^ However, some studies have questioned the validity of using the Z score, identifying inferiority when compared with the FEV_1_%.^57^ ^,^ ^58^ In view of the above and considering the various studies presented, this guideline considers that the cutoff points with the best applicability for classifying the severity of obstruction are those established by the BTA in 2002, which were therefore not modified.

In addition to FEV_1_%, the FEV_1_/FVC ratio has been used to classify the severity of OLD. Traver et al. demonstrated that it was inferior to FEV_1_% for predicting mortality, given that FVC can be reduced in individuals with severe obstruction and air trapping, paradoxically increasing this ratio.^51^ More recently, a new attempt was made to include the FEV_1_/FVC ratio in the classification of airflow obstruction severity specifically in COPD,^59^ suggesting that grading by the FEV_1_/FVC ratio is similar to the GOLD classification of severity. Considering that the classification is for the assessment of the severity of airflow obstruction by lung function and not for the prognostic evaluation of patients with COPD alone, we opted to maintain only FEV_1_% for the classification of the severity of obstruction in the present guideline.

#### Restriction: FVC

Although restriction is characterized by a reduction in TLC, few studies have evaluated the prognostic value of TLC in IPF, whereas the main clinical studies used FVC% as the primary outcome, this parameter being an independent prognostic factor, as well as being a reliable, reproducible measure that correlates well with clinical status in patients with IPF.^60^

With the aim of developing a simplified score capable of assessing the risk of mortality within 1 year, du Bois et al. evaluated data from 1,099 patients diagnosed with IPF.^61^ An analysis of FVC% showed that, in comparison with patients with an FVC% > 80%, the risk of death was 5.9 times higher among those with an FVC% < 50% (95% CI: 2.6-6.4 times), 3.6 times higher among those with an FVC% of 51-65% (95% CI: 2.0-6.5 times), and 2.2 times higher among those with an FVC% of 66-79% (95% CI: 1.2-4.1 times).

The 2005 ATS/ERS guideline suggested that only the FEV_1_% should be taken into account to classify the severity of all respiratory disorders (obstructive, restrictive, and mixed).^31^ However, that proposal has been criticized because, in fibrosing lung diseases with restriction, the FEV_1_% is better preserved by the greater elastic recoil than is the FVC, which results in an underestimation of the severity.^62^ Therefore, the FVC% should be considered to classify the severity of restriction (all cutoff points mentioned are exemplified in Table S2 in the supplementary material). Chart 7 shows the proposed classification of RLD severity, and in the present guideline, we considered the best cutoff points to be those established in the aforementioned prognostic study of IPF conducted by Du Bois et al.^61^

**Chart 7:** Cutoff values for classifying the severity of Obstructive ventilatory impairment (on the basis of the post-bronchodilator FEV_1_) and Restrictive ventilatory impairments (on the basis of the post-bronchodilator FVC), according to the recommendations of the current (2024) Brazilian Thoracic Association spirometry guidelines.

Severity	Obstruction	Restriction
	FEV_1_%	FVC%*
Mild	≥ 60	> 65-LLN
Moderate	41-59	51-65
Severe	≤ 40	≤ 50

FEV_1_%: FEV_1_ in percentage of the predicted value; and FVC%: FVC in percentage of the predicted value. *Remember that the diagnosis of restrictive lung disease is based on the reduction of TLC. In the absence of this measurement, the report should be descriptive. Note: For individuals in whom spirometry was not able to define the presence of obstructive or restrictive lung disease, there is no recommendation regarding the classification of the severity of the disorder.

### Post-BD variation

#### Initial considerations

First, to assess post-BD variation, pre- and post-BD spirometry should meet all criteria for acceptance and reproducibility. Post-BD measurements cannot be interpreted if the pre-BD spirometry is not reproducible. In such cases, the post-BD test should not be performed. The pre- and post-BD efforts should be compared. A submaximal effort can result in higher FEV_1_ values, with a false response to the bronchodilator.^63^ Therefore, it is important to always check whether the PEF is acute pre- and post-BD and whether the highest post-BD value is ≥ 90% of the pre-BD value. Special attention should be paid to the pre- and post-BD forced expiratory time, since many patients are able to prolong expiration after bronchodilator use, with an increase in FVC. Therefore, to assess a significant variation in FVC after BD use, the post-BD forced expiratory time should not exceed 10% of that obtained in the pre-BD phase.^3^

The assessment of BD response using FEV_1_ and FVC is the most commonly used method in daily practice in pulmonary function laboratories. Flows derived from the flow-volume curve and the FEF_25%-75%_ should not be considered in the assessment of BD reversibility.^26^ Flows vary with airway caliber, which in turn depends on the lung volume at which they are measured. If lung volumes change after BD use (which is common), flows should be compared at the same lung volume (isovolume), which is not usually calculated.

The role of FVC variation is well established as an aggregator of FEV_1_ variation. More severe obstruction results in greater improvement in FVC.^64^ ^,^ ^65^ In patients with pulmonary emphysema, the post-BD variation in FVC is typically greater than is the post-BD variation in FEV_1_.^66^

The use of a portable PEF meter to assess post-BD variation should be discouraged because FEF has lower sensitivity and specificity than do FEV_1_ and FVC.^67^

#### Post-BD expressions and variations

The most common ways to express post-BD variation are absolute change, percentage increase in relation to the initial spirometric value, and percentage increase in relation to the predicted value (Chart 8).

**Chart 8:** Main expressions of variations after bronchodilator use. A hypothetical example was used for better clarification.

Example: FEV_1_ = 500 mL; post-BD FEV_1_ = 600 mL; FEV_1_ = 2,500 mL		
Description	Calculation	Advantages and disadvantages
Absolute gain	(600 − 500) = 100 mL	Disadvantage: gain varies depending on sex, height, and age
Gain in % of baseline	(600 − 500) × 100 ∕ 500 = 20%	Disadvantage: gain is inversely proportional to the degree of reduction in function at baseline
Gain in % of predicted value	[600 − 500] × 100 ∕ 2500 = 4%	Advantage: minimizes the impact of differences in sex, age, height, and baseline function

BD: bronchodilator.

A large study of 4,227 adults undergoing spirometry confirmed the advantages and disadvantages of the variations presented above.^68^ Although the ATS/ERS guidelines published in 2005 considered the variation in relation to the initial value,^31^ those published in 2022 consider it in relation to the predicted value.^26^ The critique of this criterion is that it will be difficult for patients with low functional values to have a significant gain in relation to the expression in percentage of the predicted value. In the current guideline, we have maintained the recommendation to consider the variation in relation to the predicted value, which was already the expression adopted in the 2002 BTA guidelines,^3^ although it is now not considered necessary to determine the gain in the absolute value (in mL).

#### Limits of post-BD variation in functional parameters

When we are confronted with the significance of a post-BD variation, we must ask ourselves to what aspect such significance refers. This point is critical given the heterogeneity across studies, which usually differentiate between significant variation that cannot be attributed to randomness and variation that is clinically significant. These aspects are analyzed in different cohorts, in analyses of symptomatic or at-risk individuals, or even in populations without such suspicion.

Regarding the value that best expresses a post-BD variation in percentage of predicted, a study that evaluated more than 10,000 healthy individuals in 14 different countries found the upper limits of variation to be 10% for FEV_1_% and 9.2% for FVC%.^68^ Based on these findings, the ATS/ERS recently adopted as a criterion a threshold of 10% of the predicted value to indicate a significant variation in FVC or FEV_1_ in all spirometric tests, regardless of whether the results were categorized as normal or altered.^26^

A study conducted in Brazil evaluated the variation after administration of a placebo spray in 102 adult patients with airflow obstruction, with the aim of establishing the upper limits for changes in FEV_1_, vital capacity, FVC, and IC resulting from random variation. Regarding the variation in relation to the predicted values, the maximum variations (upper limits of the 95% CIs) were found to be 6% and 7% for FVC and FEV_1_, respectively.^69^ In a large cohort study, a variation of > 8% in FEV_1_% was found to be inversely associated with mortality in ill individuals and in a small proportion of healthy individuals.^70^ Therefore, we recommend that a significant post-BD variation in FEV_1_ or FVC be defined ≥ 10% of predicted for cases in which the pre-BD test result was categorized as normal and as an increase ≥ 7% of predicted for those in which it was categorized as abnormal.

The absence of significant variation after BD use does not imply that they should not be used in clinical practice, since other parameters not measured by spirometry can show reversibility. In addition, serial testing can show different values for the variation observed.

#### Post-BD variation in asthma and COPD

It is common to consider post-BD variation useful for making the distinction between asthma and COPD. One aspect of asthma is variability in lung function, bronchial hyperresponsiveness, and significant post-BD variation, the last being one of the criteria for the diagnosis of asthma established in the GINA and ERS guidelines.^71^ ^,^ ^72^ However, in individuals with asthma, the spirometry results can be normal or show no significant post-BD variation, although neither condition excludes the diagnosis. Similarly, the idea that the absence of post-BD variation is necessary for the diagnosis of COPD is mistaken, since such variation is often present even in the absence of associated asthma. Therefore, the use of post-BD variation as the sole tool to differentiate between asthma and COPD is not recommended.^73^ ^-^ ^75^ Patients with COPD who present a post-BD variation in FVC alone have more symptoms and worse lung function.^64^

#### Recommended bronchodilator and the interval between its administration and the post-BD testing

Given the availability and widespread use of albuterol, it is the recommended bronchodilator. It should be used at a dose of 400 µg, with a 15-min wait before performing the post-BD test.

## FINAL CONSIDERATIONS

Spirometry is an essential diagnostic tool for any individual with or at risk of developing lung disease and is also a functional measurement to be used in individuals with higher-risk conditions, such as those who have undergone lung resection. For a correct diagnosis, it is essential that the spirometry findings be interpreted in conjunction with the clinical context and pretest probability for the individual. This concept becomes even more relevant for tests with borderline alterations. In this context, we suggest that the report not be limited to the functional diagnosis of obstruction or restriction and that expressions such as “correlate with clinical context or pretest probability” be used.

The choice of predicted values is important. Despite attempts by international initiatives to standardize the use of the GLI equation, we have seen significant differences in relation to other equations, mainly in that the GLI equation fails to characterize the presence of obstruction because it uses LLN values for the FEV_1_/FVC ratio that are low in comparison with those predicted for the population of Brazil. It is of fundamental importance that new studies be conducted in order to update the reference values for adolescents, as well as for young women and men under 20 and 25 years of age, respectively.

Finally, the recommendation to characterize significant variation in bronchodilator response sought to adopt different limits for individuals with normal results on the initial spirometry tests than for those with functional abnormalities, because of the difference in the degree of response between those two populations.

## References

[1] Miller MR, Hankinson J, Brusasco V, Burgos F, Casaburi R, Coates A (2005). Standardisation of spirometry. Eur Respir J.

[2] Graham BL, Steenbruggen I, Miller MR, Barjaktarevic IZ, Cooper BG, Hall GL (2019). Standardization of Spirometry 2019 Update An Official American Thoracic Society and European Respiratory Society Technical Statement. Am J Respir Crit Care Med.

[3] Sociedade Brasileira de Pneumologia e Tisiologia (2002). Diretrizes para Testes de Função Pulmonar. J Pneumol.

[4] Welsh EJ, Bara A, Barley E, Cates CJ (2010). Caffeine for asthma. Cochrane Database Syst Rev.

[5] Duffy P, Phillips YY (1991). Caffeine consumption decreases the response to bronchoprovocation challenge with dry gas hyperventilation. Chest.

[6] Yurach MT, Davis BE, Cockcroft DW (2011). The effect of caffeinated coffee on airway response to methacholine and exhaled nitric oxide. Respir Med.

[7] Mottram CD (2012). Ruppel's Manual of Pulmonary Function Testing..

[8] Pereira CAC (2021). Testes de Função Pulmonar - Bases, Interpretações e Aplicações Clínicas..

[9] Townsend MC (1984). Spirometric forced expiratory volumes measured in the standing versus the sitting posture. Am Rev Respir Dis.

[10] Bucca CB, Carossa S, Colagrande P, Brussino L, Chiavassa G, Pera P (2001). Effect of edentulism on spirometric tests. Am J Respir Crit Care Med.

[11] Indrakumar HS, Venkatesh D, Adoni VV, Kashyap R, Jayanthi D, Prakash N (2018). Spirometric Assessment of Impact of Complete Dentures on Respiratory Performance An in vitro Study. J Contemp Dent Pract.

[12] Rodrigues R, Berton DC, Neder JA (2023). Padronização Técnica dos Principais Testes de Função Pulmonar:.

[13] Sylvester KP, Clayton N, Cliff I, Hepple M, Kendrick A, Kirkby J (2020). ARTP statement on pulmonary function testing 2020. BMJ Open Respir Res.

[14] Cooper BG (2011). An update on contraindications for lung function testing. Thorax.

[15] Sharifi V, Brazzale DJ, Lanteri CJ, Ruehland WR (2023). The Effect of 2019 American Thoracic Society/European Respiratory Society Criteria for Back-extrapolated Volume on the Acceptability and Interpretation of Spirometry Results Is Minor. Ann Am Thorac Soc.

[16] Hegewald MJ, Lefor MJ, Jensen RL, Crapo RO, Kritchevsky SB, Haggerty CL (2007). Peak expiratory flow is not a quality indicator for spirometry peak expiratory flow variability and FEV1 are poorly correlated in an elderly population. Chest.

[17] Grasbeck R (2004). The evolution of the reference value concept. Clin Chem Lab Med.

[18] Bhakta NR, Bime C, Kaminsky DA, McCormack MC, Thakur N, Stanojevic S, Baugh AD (2023). Race and Ethnicity in Pulmonary Function Test Interpretation An Official American Thoracic Society Statement. Am J Respir Crit Care Med.

[19] Jones RL, Nzekwu MM (2006). The effects of body mass index on lung volumes. Chest.

[20] Gibson GJ (2009). Normal Variation. Clinical Tests of Respiratory Function, 3th Ed.

[21] Labaki WW (2024). FEV(1) More than a Measurement of Lung Function, A Biomarker of Health. Am J Respir Crit Care Med.

[22] Miller MR, Thinggaard M, Christensen K, Pedersen OF, Sigsgaard T (2014). Best lung function equations for the very elderly selected by survival analysis. Eur Respir J.

[23] Pereira CA, Sato T, Rodrigues SC (2007). New reference values for forced spirometry in white adults in Brazil. J Bras Pneumol.

[24] Quanjer PH, Stanojevic S, Cole TJ, Baur X, Hall GL, Culver BH (2012). Multi-ethnic reference values for spirometry for the 3-95-yr age range the global lung function 2012 equations. Eur Respir J.

[25] Pereira CA, Duarte AA, Gimenez A, Soares MR (2014). Comparison between reference values for FVC, FEV1, and FEV1/FVC ratio in White adults in Brazil and those suggested by the Global Lung Function Initiative 2012. J Bras Pneumol.

[26] Stanojevic S, Kaminsky DA, Miller MR, Thompson B, Aliverti A, Barjaktarevic I (2022). ERS/ATS technical standard on interpretive strategies for routine lung function tests. Eur Respir J.

[27] Prata TA, Mancuzo E, Pereira CAC, Miranda SS, Sadigursky LV, Hirotsu C (2018). Spirometry reference values for Black adults in Brazil. J Bras Pneumol.

[28] Jones MH, Vidal PCV, Lanza FC, Silva D, Pitrez PM, Olmedo A (2020). Reference values for spirometry in Brazilian children. J Bras Pneumol.

[29] Miller MR, Quanjer PH, Swanney MP, Ruppel G, Enright PL (2011). Interpreting lung function data using 80% predicted and fixed thresholds misclassifies more than 20% of patients. Chest.

[30] Global Initiative for Chronic Obstructive Lung Disease (GOLD) [homepage on the Internet] Global Strategy for Prevention, Diagnosis and Management of COPD: 2024.

[31] Pellegrino R, Viegi G, Brusasco V, Crapo RO, Burgos F, Casaburi R (2005). Interpretative strategies for lung function tests. Eur Respir J.

[32] Bhatt SP, Balte PP, Schwartz JE, Cassano PA, Couper D, Jacobs DR (2019). Discriminative Accuracy of FEV1 FVC Thresholds for COPD Related Hospitalization and Mortality. JAMA.

[33] Buhr RG, Barjaktarevic IZ, Quibrera PM, Bateman LA, Bleecker ER, Couper DJ (2022). Reversible Airflow Obstruction Predicts Future Chronic Obstructive Pulmonary Disease Development in the SPIROMICS Cohort An Observational Cohort Study. Am J Respir Crit Care Med.

[34] Chhabra SK (1998). Forced vital capacity, slow vital capacity, or inspiratory vital capacity which is the best measure of vital capacity?. J Asthma.

[35] Saint-Pierre M, Ladha J, Berton DC, Reimao G, Castelli G, Marillier M (2019). Is the Slow Vital Capacity Clinically Useful to Uncover Airflow Limitation in Subjects With Preserved FEV(1)/FVC Ratio. Chest.

[36] Fortis S, Comellas AP, Bhatt SP, Hoffman EA, Han MK, Bhakta NR (2021). Ratio of FEV(1)/Slow Vital Capacity of &lt; 0 7 Is Associated With Clinical, Functional, and Radiologic Features of Obstructive Lung Disease in Smokers With Preserved Lung Function. Chest.

[37] Dykstra BJ, Scanlon PD, Kester MM, Beck KC, Enright PL (1999). Lung volumes in 4,774 patients with obstructive lung disease. Chest.

[38] Pereira C, Sato T (1991). Limitação ao fluxo aéreo e capacidade vital reduzida distúrbio ventilatório obstrutivo ou combinado. J Pneumol.

[39] Wong BMS, Silva AMD, Maurici R, Melo JT (2023). Pathophysiology of reduced forced vital capacity with airflow obstruction on spirometry performance of two mathematical models in clinical practice. J Bras Pneumol.

[40] Knox-Brown B, Mulhern O, Feary J, Amaral AFS (2022). Spirometryparameters used to define small airways obstruction in population based studies systematic review. Respir Res.

[41] Quanjer PH, Weiner DJ, Pretto JJ, Brazzale DJ, Boros PW (2014). Measurement of FEF25-75% and FEF75% does not contribute to clinical decision making. Eur Respir J.

[42] Ronish BE, Couper DJ, Barjaktarevic IZ, Cooper CB, Kanner RE, Pirozzi CS (2022). Forced Expiratory Flow at 25%-75% Links COPD Physiology to Emphysema and Disease Severity in the SPIROMICS Cohort. Chronic Obstr Pulm Dis.

[43] Knox-Brown B, Potts J, Santofimio VQ, Minelli C, Patel J, Abass NM (2023). Isolated small airways obstruction predicts future chronic airflow obstruction: a multinational longitudinal study. BMJ Open Respir Res.

[44] Gelb AF, Yamamoto A, Verbeken EK, Hogg JC, Tashkin DP, Tran DNT (2021). Normal Routine Spirometry Can Mask COPD/Emphysema in Symptomatic Smokers. Chronic Obstr Pulm Dis.

[45] Hansen JE, Sun XG, Wasserman K (2006). Discriminating measures and normal values for expiratory obstruction. Chest.

[46] Swanney MP, Beckert LE, Frampton CM, Wallace LA, Jensen RL, Crapo RO (2004). Validity of the American Thoracic Society and other spirometric algorithms using FVC and forced expiratory volume at 6 s for predicting a reduced total lung capacity. Chest.

[47] D'Aquino LC, Rodrigues SC, Barros JA, Rubin AS, Rosario NA, Pereira CA (2010). Predicting reduced TLC in patients with low FVC and a normal or elevated FEV1/FVC ratio. J Bras Pneumol.

[48] McGinn EA, Mandell EW, Smith BJ, Duke JW, Bush A, Abman SH (2023). Dysanapsis as a Determinant of Lung Function in Development and Disease. Am J Respir Crit Care Med.

[49] Dos Santos Andreata L, Soares MR, Pereira CA (2019). Reduced FEV(1)/FVC and FEV(1) in the Normal Range as a Physiological Variant. Respir Care.

[50] Anthonisen NR, Wright EC, Hodgkin JE (1986). Prognosis in chronic obstructive pulmonary disease. Am Rev Respir Dis.

[51] Traver GA, Cline MG, Burrows B (1979). Predictors of mortality in chronic obstructive pulmonary disease A 15-year follow-up study. Am Rev Respir Dis.

[52] Kanner RE, Renzetti AD, Stanish WM, Barkman HW, Klauber MR (1983). Predictors of survival in subjects with chronic airflow limitation. Am J Med.

[53] Esteban C, Quintana JM, Egurrola M, Moraza J, Aburto M, Perez-Izquierdo J (2009). Classifying the severity of COPD are the new severity scales better than the old?. Int J Tuberc Lung Dis.

[54] (1997). BTS guidelines for the management of chronic obstructive pulmonary disease. The COPD Guidelines Group of the Standards of Care Committee of the BTS. Thorax.

[55] (1995). Standards for the diagnosis and care of patients with chronic obstructive pulmonary disease American Thoracic Society. Am J Respir Crit Care Med.

[56] Almagro P, Martinez-Camblor P, Soriano JB, Marin JM, Alfageme I, Casanova C (2014). Finding the best thresholds of FEV1 and dyspnea to predict 5-year survival in COPD patients the COCOMICS study. PLoS One.

[57] Tejero E, Prats E, Casitas R, Galera R, Pardo P, Gavilan A (2017). Classification of Airflow Limitation Based on z-Score Underestimates Mortality in Patients with Chronic Obstructive Pulmonary Disease. Am J Respir Crit Care Med.

[58] Huang TH, Hsiue TR, Lin SH, Liao XM, Su PL, Chen CZ (2018). Comparison of different staging methods for COPD in predicting outcomes. Eur Respir J.

[59] Bhatt SP, Nakhmani A, Fortis S, Strand MJ, Silverman EK, Sciurba FC (2023). FEV(1)/FVC Severity Stages for Chronic Obstructive Pulmonary Disease. Am J Respir Crit Care Med.

[60] du Bois RM, Weycker D, Albera C, Bradford WZ, Costabel U, Kartashov A (2011). Forced vital capacity in patients with idiopathic pulmonary fibrosis test properties and minimal clinically important difference. Am J Respir Crit Care Med.

[61] du Bois RM, Weycker D, Albera C, Bradford WZ, Costabel U, Kartashov A (2011). Ascertainment of individual risk of mortality for patients with idiopathic pulmonary fibrosis. Am J Respir Crit Care Med.

[62] Aggarwal AN, Agarwal R (2007). The new ATS/ERS guidelines for assessing the spirometric severity of restrictive lung disease differ from previous standards. Respirology.

[63] Krowka MJ, Enright PL, Rodarte JR, Hyatt RE (1987). Effect of effort on measurement of forced expiratory volume in one second. Am Rev Respir Dis.

[64] Barjaktarevic IZ, Buhr RG, Wang X, Hu S, Couper D, Anderson W (2019). Clinical Significance of Bronchodilator Responsiveness Evaluated by Forced Vital Capacity in COPD SPIROMICS Cohort Analysis. Int J Chron Obstruct Pulmon Dis.

[65] Quanjer PH, Ruppel GL, Langhammer A, Krishna A, Mertens F, Johannessen A (2017). Bronchodilator Response in FVC Is Larger and More Relevant Than in FEV(1) in Severe Airflow Obstruction. Chest.

[66] Han MK, Wise R, Mumford J, Sciurba F, Criner GJ, Curtis JL (2010). Prevalence and clinical correlates of bronchoreversibility in severe emphysema. Eur Respir J.

[67] Aggarwal AN, Agarwal R, Gupta D, Jindal SK (2009). Use of peak expiratory flow for assessing bronchodilator responsiveness. Prim Care Respir J.

[68] Tan WC, Vollmer WM, Lamprecht B, Mannino DM, Jithoo A, Nizankowska-Mogilnicka E (2012). Worldwide patterns of bronchodilator responsiveness results from the Burden of Obstructive Lung Disease study. Thorax.

[69] Soares AL, Pereira CA, Rodrigues SC (2013). Spirometric changes in obstructive disease after all, how much is significant?. J Bras Pneumol.

[70] Ward H, Cooper BG, Miller MR (2015). Improved criterion for assessing lung function reversibility. Chest.

[71] Louis R, Satia I, Ojanguren I, Schleich F, Bonini M, Tonia T (2022). European Respiratory Society Guidelines for the Diagnosis of Asthma in Adults. Eur Respir J.

[72] Levy ML, Bacharier LB, Bateman E, Boulet LP, Brightling C, Buhl R (2023). Key recommendations for primary care from the 2022 Global Initiative for Asthma (GINA) update. NPJ Prim Care Respir Med.

[73] Janson C, Malinovschi A, Amaral AFS, Accordini S, Bousquet J, Buist AS (2019). Bronchodilator reversibility in asthma and COPD: findings from three large population studies. Eur Respir J.

[74] Chhabra SK (2005). Acute bronchodilator response has limited value in differentiating bronchial asthma from COPD. J Asthma.

[75] Tuomisto LE, Ilmarinen P, Lehtimaki L, Tommola M, Kankaanranta H (2019). Immediate bronchodilator response in FEV(1) as a diagnostic criterion for adult asthma. Eur Respir J.

